# The risk of psychosis for transgender individuals: a Dutch national cohort study

**DOI:** 10.1017/S0033291723002088

**Published:** 2023-12

**Authors:** Fabian Termorshuizen, Annelou L.C. de Vries, Chantal M. Wiepjes, Jean-Paul Selten

**Affiliations:** 1 Rivierduinen Institute for Mental Health Care, Sandifortdreef 19, 2333 ZZ Leiden, the Netherlands; 2 Department of Psychiatry and Neuropsychology, School for Mental Health and Neuroscience, Maastricht University Medical Center, P.O. Box 616, 6200 MD Maastricht, the Netherlands; 3 Department of Child and Adolescent Psychiatry, Center of Expertise on Gender Dysphoria, Amsterdam University Medical Centers, Vrije Universiteit, Amsterdam, the Netherlands; 4 Department of Endocrinology, Center of Expertise on Gender Dysphoria, Amsterdam University Medical Centers, Vrije Universiteit, Amsterdam, the Netherlands

**Keywords:** Epidemiology, minority stress, psychotic disorder, social defeat, transgender persons

## Abstract

**Background:**

The stressful minority position of transgender persons may result in a high risk of psychosis. Conflicting data suggest that the observed risk depends on setting of recruitment. We assessed the relative risk of non-affective psychotic disorder (NAPD) in a large, representative cohort of transgender persons.

**Methods:**

This cohort was composed using: data on legal sex change from the Dutch population registry and data on dispensing of cross-sex hormones (route 1), and a registry of insurance claims from mental health care including persons with a diagnosis of gender identity disorder (DSM-IV) or gender dysphoria (DSM-5) (route 2). They were matched by sex at birth, calendar year and country of birth to controls from the general population. Transgender persons (*N* = 5564) and controls (*N* = 27 820), aged 16–60 years at 1 January 2011, were followed until the first insurance claim for NAPD in 2011–2019.

**Results:**

The incidence rate ratio (IRR) of NAPD for transgender persons selected exclusively through route 1 (*N* = 3859, IRR = 2.00, 95%-CI 1.52–2.63) was increased, but significantly lower than the IRRs for those selected exclusively through route 2 (*N* = 694, IRR = 22.15, 95%-CI 13.91–35.28) and for those found by both routes (*N* = 1011, IRR = 5.17, 95%-CI 3.57–7.49; *p* value for differences in IRR < 0.001).

**Conclusions:**

This study supports the social defeat-hypothesis of NAPD. The results also show the presence of a substantial number of transgender persons with severe psychiatric problems who have not (yet) taken steps to gender-affirmative care.

## Introduction

Transgender persons experience a marked discrepancy between their sex assigned at birth and their gender identity and many of them will opt for gender-affirmative treatment at a specialised gender identity clinic (Coleman *et al*. 2012). They often suffer from this discrepancy, but also from frequently co-occurring psychiatric disorders (Brown & Jones, 2016; de Freitas, Leda-Rego, Bezerra-Filho, & Miranda-Scippa, 2020; Dhejne, Van Vlerken, Heylens, & Arcelus, 2016; Wiepjes *et al*. 2020).

Given their frequent experiences of discrimination and stigmatisation one may ask the question as to whether they are at an increased risk of non-affective psychotic disorder (NAPD) (Bazargan & Galvan, 2012; Pellicane & Ciesla, 2022). An increased risk of NAPD has been reported for many other socially disadvantaged groups, including migrants from non-Western countries in Europe (Selten, van der Ven, & Termorshuizen, 2020), African-Americans (Bresnahan *et al*. 2007), Australian Aboriginals (Mirza *et al*. 2022), Maori in New Zealand (Petrovic-van der Deen *et al*. 2020), those with a hearing impairment (Linszen, Brouwer, Heringa, & Sommer, 2016), a homosexual orientation (Bolton & Sareen, 2011; Post, Veling, & Group investigators, 2021), an autism spectrum disorder (Schalbroeck, Termorshuizen, Visser, van Amelsvoort, & Selten, 2019), or a low IQ (Khandaker, Barnett, White, & Jones, 2011). According to the social defeat hypothesis of NAPD, the experience of an outsider status or a subordinate position may lead to dopamine dysregulation and thereby contribute to an increased risk for these very different populations (Selten & Cantor-Graae, 2005; Selten & Ormel, 2023; Selten, van der Ven, Rutten, & Cantor-Graae, 2013).

A number of studies from specialised gender identity clinics did not report a substantially increased risk of psychosis for transgender persons (Cole, O'Boyle, Emory, & Meyer, 1997; Gomez-Gil, Trilla, Salamero, Godas, & Valdes, 2009; Haraldsen & Dahl, 2000; Hepp, Kraemer, Schnyder, Miller, & Delsignore, 2005; Heylens *et al*. 2014; Mazaheri Meybodi, Hajebi, & Ghanbari Jolfaei, 2014; Simonsen, Giraldi, Kristensen, & Hald, 2016). The recruitment at these clinics, however, probably involves a selection of a relatively healthy psychosis-free group. This is understandable, because clinicians are concerned about an unfavourable outcome of a gender-affirmative treatment in individuals with mental illness (Dhejne *et al*. 2011; Meijer, Eeckhout, van Vlerken, & de Vries, 2017). Furthermore, misdiagnoses of psychotic states as gender identity disorder (GID) or gender dysphoria (GD) have been reported (À Campo, Nijman, Merckelbach, & Evers, 2003). In recent years, a number of studies using large databases with health care records from hospitals and insurance companies showed a substantially increased risk of many psychiatric disorders, including psychosis (Barr, Roberts, & Thakkar, 2021; Dragon, Guerino, Ewald, & Laffan, 2017; Hanna *et al*. 2019). However, it is not clear from these studies in which treatment setting the diagnosis of GID/GD was established: gender identity clinics, departments of internal medicine or surgery, or mental health care.

The present study from the Netherlands compared the risk of NAPD among transgender persons to that for the general population. In order to recruit a large and representative sample (thus, not restricted to those treated at a gender identity clinic), we used several data sources: national record-linked population-based data on administrative change of legal sex, data on dispensing of cross-sex hormones and data on insurance claims from institutes for mental health care (iMHC). Given the frequent co-occurring of psychiatric disorders and reluctance of physicians to deliver gender-affirmative care to those with a mental illness, we hypothesised an increased risk of NAPD among transgender persons, in particular for those with a registered DSM-IV diagnosis of GID or a DSM-5 diagnosis of GD from an iMHC, but without evidence of administrative or hormonal gender affirmation. In addition, we estimated the relative risks of psychosis for transgender persons assigned the male sex at birth (AMAB) and for those assigned the female sex at birth (AFAB) separately. This was done to examine whether the reported finding of the high relative risk of psychosis for men compared to women is also observed for transgender persons, either on the basis of sex assigned at birth or on the basis of experienced gender identity (Castillejos, Martin-Perez, & Moreno-Kustner, 2018).

## Methods

### Data sources

The Dutch population register managed by Statistics Netherlands (Centraal Bureau voor de Statistiek, CBS) records information on the date of birth, legal sex, (parental) country of birth, address of residence, dates of immigration and emigration and date of death of all legal residents in the Netherlands. To ascertain someone's sex at birth, additional information on administrative change in legal sex was record-linked to these data.

The second database, run by the Health Care Institute Netherlands (Zorginstituut Nederland, ZiN), provides information on dispensed medication reimbursed by health-insurance companies during the period 2006–2020. This database records information on drugs dispensed to outpatients and to patients in nursing homes, but not to in-patients. As for the present study, the first four positions of the ATC code are mentioned only once for a particular calendar year and an insured individual. Thus, it is possible to establish whether an individual has ever received a drug from a certain class (e.g. N05A: antipsychotic medication) in a certain calendar year.

The third database is the register of the so-called Diagnosis Treatment Combinations (DTCs) (Dutch: Diagnose Behandel Combinatie, DBC) of the Dutch Healthcare Authority (Nederlandse Zorgautoriteit, NZa). The NZa collects information from all health-insurance companies in the Netherlands. A DTC is an insurance claim, that has to be renewed each year, based on codes for diagnosis and treatment by a medical specialist, with accompanying starting- and end-dates. For the present study, DTC data from all iMHC in the Netherlands were available for the calendar years 2011–2019. These claims do not cover psychiatric counselling services in general hospitals. Patients with chronic psychiatric problems, however, will almost always be referred to an iMHC.

Staff of Statistics Netherlands linked the information from the three databases, using the civil identification number, unique for each Dutch citizen. Dutch privacy laws allow the use of personal (health care) data for medical-scientific research without informed consent, provided that the results of the analysis cannot be traced to an individual. Consequently, the postal code and the civil identification number were removed from the files used in this study.

### Study group

The study group was selected using different strategies. First, subjects were selected using information on dispensed sex hormones opposite to their sex at birth (2006–2020). For those AMAB, this is oestrogen (ATC code: G03C) with or without anti-androgens or GnRH analogues (ATC codes: G03H or L02A). For those AFAB, this is androgens (ATC code: G03B). To avoid the inclusion of men with prostate carcinoma or women treated with testosterone for (peri)menopausal symptoms, subjects aged 45 years or older at the time of the first dispensing and without other information indicative of a transgender status were placed in a category ‘uncertain’. Secondly, individuals were selected for whom an administrative change in legal sex was registered in the years 1997–2020. Thirdly, persons with an insurance claim from an iMHC for GID/GD, either as main or second diagnosis (DSM-IV or DSM-5 codes: 302.85 and 302.6), were selected. This selection was restricted to the years 2011–2016, because these diagnoses were placed in a broad category ‘other’ from 2017 onwards.

The study group was categorised by three non-overlapping routes of selection: those found by dispensing of cross-sex hormones and/ or administrative change in legal sex, but not by an insurance claim for GID/GD from an iMHC (route 1), those found by an insurance claim, but not by dispensing of cross-sex hormones and not by administrative change in legal sex (route 2), and those found by both (i) dispensing of cross-sex hormones and/ or administrative change in sex and (ii) an insurance claim for GID/GD (overlap: route 3). This categorisation was used to distinguish between transgender persons with (routes 1 and 3) and those without steps towards gender affirmation (route 2), and to distinguish those with (routes 2 and 3) and without a registered diagnosis of GID/GD from an iMHC (route 1).

The final analyses included only those who were legal residents in the Netherlands and alive during (a part of) the period 2011–2019, which was the time interval for assessment of a NAPD (see below). Furthermore, the study group for the final analysis was restricted to persons aged 16–60 years at January 1, 2011.

For each transgender person, five controls from the general population were selected with identical inclusion criteria, matched at an individual level by sex at birth, calendar year of birth (in 5-years categories), country of origin, and migration status (1st v. 2nd generation, see online Supplement). This matching strategy necessarily implies a prevalence of transgender status of 20% in the final study sample, which is not a representative figure for the source population. The pool of controls consisted of all citizens of the Netherlands with legal residence for whom no indication of a possible transgender status was found in the used registries. Transgender persons and controls were matched by sex at birth, not by experienced gender, as sex at birth is an objective fact with a very low risk of misclassification. Moreover, a comparison of transgender persons with controls matched by (presumed) gender experience implies a comparison across two characteristics: sex at birth and transgender status.

In order to examine whether those with a diagnosis of GID/GD from an iMHC (that is, selected by route 2 or route 3) are at a higher risk of psychosis than other persons treated at an iMHC, we composed a second group of controls with an identical matching procedure, consisting of individuals with an insurance claim from an iMHC regardless of diagnosis (except GID/GD).

### Follow-up

Both transgender persons and matched controls were followed for the development of NAPD. Follow-up started at 1 January 2011 and ended at 31 December 2019. This is the period for which information on DTCs was available and treatment for NAPD at an iMHC could be established. Follow-up ended in the calendar year of the first registered DTC for NAPD or when the person died. Periods following emigration or before immigration were excluded from the individual follow-up time.

### Outcome: DTC for NAPD

For each individual and calendar year of follow-up, we checked whether a DTC with a diagnostic code for NAPD was present. That is, all DSM-IV codes indicative of NAPD, either as main or as second diagnosis, were selected (295.xx, 297.1/3, 298.8/9). The first DTC for NAPD during the period 2011–2019 was the primary outcome and regarded as the incident moment within the time frame of observation. DTCs for NAPD and follow-up time after the first DTC were excluded from the analysis. For those with NAPD, data on dispensing of antipsychotic medication (ATC code N05A) during the 5 years before the first DTC for NAPD were linked to the treatment record. This was done to find a possible earlier moment of treatment and to re-define the incident moment of NAPD.

### Statistical analysis

For each stratum (defined by transgender status, route of selection, and sex at birth), the numbers of DTCs for NAPD and days of follow-up were calculated. The number of DTCs divided by follow-up time was used as the outcome in a Poisson regression analysis. In the first analysis, the incidence rate ratio (IRR) of treatment for NAPD among transgender persons compared to controls was estimated by route of selection. Terms for interaction of {transgender status x route} were included to find out whether the IRR differed by route. For transgender persons found by routes 2 and 3, the analysis was repeated using controls with a treatment record at an iMHC (‘iMHC controls’). Next, the analyses were stratified by sex at birth, that is, transgender persons AMAB v. transgender persons AFAB.

In a sensitivity analysis, the incident moment of NAPD was re-defined as the first calendar year of a dispensing of antipsychotic medication within the 5 years prior to the calendar year of the first DTC for NAPD. Cases of NAPD who had received antipsychotic medication before 2011 were excluded from this analysis. The re-defined ‘incident moment’ was used to arrive at figures that better approximate the life-time incidence and life-time IRRs.

Data preparation, record linkage and estimation of crude rates were performed using SPSS version 25.0. The Poisson regression analysis was conducted using STATA version 16.0.

## Results

### Identification of transgender persons, baseline characteristics

Table 1 gives details on putative transgender persons by route of selection. The numbers of transgender persons selected using routes 1 (that is, identified by legal sex change and/ or dispensing of cross-sex hormones), 2 (that is, identified by a registered diagnosis of GID/GD at an iMHC), and 3 (that is, identified by (i) legal sex change and/ or dispensing of cross-sex hormones and (ii) a diagnosis of GID/GD at an iMHC) were 3589, 694, and 1011, respectively. We placed 1322 persons with a possible transgender status in the category ‘uncertain’ (see Methods). Table 2 shows the numbers by sex at birth, age, and migration status for those selected by routes 1, 2, 3, and for their matched controls.

**Table 1.** Selection of transgender persons in the Netherlands using information on administrative change of legal sex, dispensing of cross-sex hormones, and on registered diagnoses of gender identity disorder (DSM-IV) or gender dysphoria (DSM-5) at an institute for mental health care

Administrative change in legal sex	Dispensing of hormones opposite to sex assigned at birth	Diagnosis of GID/GD^c^ from an iMHC^d^	Route of selection^e^	Numbers in registries	Numbers that met inclusion criteria^f^
No	AMAB^a^: oestrogen & anti-androgen, <45 year^g^	No	1	459	302
No	AMAB: oestrogen, <45 year	No	1	477	215
No	AFAB^b^: androgen, <45 year	No	1	876	691
Yes AMAB	No	No	1	246	121
Yes AMAB	AMAB: oestrogen & anti-androgen	No	1	1764	1248
Yes AMAB	AMAB: oestrogen	No	1	305	235
Yes AFAB	No	No	1	187	51
Yes AFAB	AFAB: androgen	No	1	2132	996
Total N route 1				+6446	+3859
No	No	Yes	2	972	694
Total N route 2				+972	+694
No	AMAB: oestrogen & anti-androgen	Yes	3	112	94
No	AMAB: oestrogen	Yes	3	17	15
No	AFAB: androgen	Yes	3	45	31
Yes AMAB	No	Yes	3	15	13
Yes AMAB	AMAB: oestrogen & anti-androgen	Yes	3	600	488
Yes AMAB	AMAB: oestrogen	Yes	3	39	38
Yes AFAB	AFAB: androgen	Yes	3	512	332
Total N route 3				+1340	+1011
No	AMAB?: oestrogen & anti-androgen, >45 year^h^	No	Uncertain	1045	126
No	AMAB?: oestrogen, >45 year	No	Uncertain	1010	262
No	AFAB?: androgen, >45 year	No	Uncertain	1412	934
Total N uncertain				+3467	+1322
Overall Total				+12 225	+6886

^a^AMAB, Transgender person assigned the male sex at birth; ^b^AFAB, Transgender person assigned the female sex at birth; ^c^ GID, gender identity disorder, according to DSM-IV criteria, GD, gender dysphoria, according to similar DSM-5 criteria; ^d^iMHC, institute for mental health care; ^e^Routes of selection, Route 1: administrative change in legal sex and/ or dispensing of cross-sex hormones, Route 2: diagnosis of gender identity disorder established at an institute for mental health care, Route 3: overlap between Route 1 and 2; ^f^inclusion criteria, alive and legal residence in the Netherlands during period 2011–2019 and aged 16–60 years at January 1, 2011; ^g^No administrative change in legal sex or diagnosis of gender identity disorder from an institute for mental health care. In order to render transgender status very likely, subjects who used cross-sex hormones were required to be younger than 45 years at the time of first dispensing of these hormones. ^h^No administrative change in legal sex or diagnosis of gender identity disorder from an institute for mental health care. Since a number of these individuals might use cross-sex hormones for other purposes than transgender status, they were placed in the category ‘uncertain’.

**Table 2.** Baseline characteristics of transgender persons in the Netherlands and controls from the general population, by route of recruitment

	Transgender persons Route 1		Controls		Transgender persons Route 2		Controls		Transgender persons Route 3		Controls	
Number	3859		19 295		694		3470		1011		5055	
Sex at birth(No,%): Male	2121	55.0	10 605	55.0	458	66.0	2290	66.0	648	64.1	3240	64.1
Female	1738	45.0	8690	45.0	236	34.0	1180	34.0	363	35.9	1815	35.9
Age at 1/1/2011(No,%):												
>16 - <45 year	3164	82.0	15 757	81.7	509	73.3	2567	74.0	831	82.2	4156	82.2
>45 year - <60 year	695	18.0	3538	18.3	185	26.7	903	26.0	180	17.8	899	17.8
Migration status (No,%)												
Dutch	2710	70.2	13 550	70.2	507	73.1	2535	73.1	765	75.7	3825	75.7
Non-Western 1st gen.^1^	560	14.5	2800	14.5	59	8.5	295	8.5	89	8.8	445	8.8
Non-Western 2nd gen.^2^	263	6.8	1315	6.8	62	8.9	310	8.9	73	7.2	365	7.2
Western^3^ 1st gen.	182	4.7	910	4.7	32	4.6	160	4.6	37	3.7	185	3.7
Western^4^ 2nd gen.	144	3.7	720	3.7	34	4.9	170	4.9	47	4.6	235	4.6

^1^1st gen., first-generation migrant; ^2^2nd gen., second-generation migrant; ^3^Non-Western, all other countries not mentioned under^4, 4^Western, Europe, countries of the former Soviet Union with a predominantly Christian religion, the USA, Canada, Australia, New-Zealand.

### Risk of NAPD by route of selection

Table 3 gives the numbers of persons, person-years of follow-up, and DTCs for NAPD, associated rates, and the IRR of NAPD for transgender persons compared to controls, by route of selection. The incidence rates of NAPD were significantly higher for transgender persons than for matched controls, for each route of selection. However, the differences in the IRR by route of selection were large. The IRR for transgender persons selected via route 2 was extremely high (IRR = 22.15, 95%-CI 13.91–35.28). When we restricted the group of controls to those with a registered DTC from an iMHC, this IRR dropped substantially, but it remained significantly higher than 1.00 (IRR = 1.95, 95%-CI 1.53–2.47). The IRR for transgender persons selected via route 3 compared to controls with a registered DTC from an iMHC was non-significantly lower than 1.00 (IRR = 0.88, 95%-CI 0.66–1.16). However, these transgender persons could also have been identified without any information from iMHC. Thus, matching to controls from iMHC may be regarded as ‘overmatching’ and the resulting IRR probably an underestimation of the true IRR.

**Table 3.** Transgender persons in the Netherlands v. controls from the general population and (for route 2 and 3) controls from mental health care: numbers of persons, numbers of person-years of follow-up, numbers of Diagnosis Treatment Combinations for non-affective psychotic disorder (DTCs for NAPD, 2011–2019), Rates (number/ 10 000 person-years), and incidence rate ratios (IRRs), by route of recruitment (see Table 1)

	Controls				Transgender persons					
	N	Number of person-years	Nr of DTCs for NAPD	Rate	N	Number of person-years	Nr of DTCs for NAPD	Rate	IRR	95%-confidence interval
	Controls from the general population									
Route										
1	19 295	156 730	175	11.2	3859	32 222	72	22.4	2.00	1.52–2.63
2	3470	28 971	22	7.6	694	5469	92	168.2	22.15	13.91–35.28
3	5055	42 212	55	13.0	1011	8456	57	67.4	5.17	3.57–7.49
						route 1,2, v. 3: *χ*2, df, *p* value			78.29, 2	<0.001
Uncertain	6610	56 208	44	7.8	1322	11 349	13	11.5	1.48	0.79–2.72
	Controls from mental health care									
Route										
2	3470	28 350	245	86.4	694	5469	92	168.2	1.95	1.53–2.47
3	5055	41 902	322	76.9	1011	8456	57	67.4	0.88	0.66–1.16
						route 1,2, v. 3: *χ*2, df, *p* value			22.49, 2	<0.001

Similar results were obtained when data on dispensed antipsychotic medication was used to re-define the incident moment of treatment for NAPD (online Supplementary Table 1).

Similar IRRs were found when we restricted the analysis to those under the age of 25 years at January 1, 2011, which suggests that our findings are also relevant for lifetime-incident cases of NAPD (data available on request).

### Risk of NAPD by route of selection and sex at birth

Table 4 shows that within each route of selection the IRRs were significantly increased for both transgender persons AMAB and transgender persons AFAB. For those AFAB, however, the IRRs were substantially higher than those AMAB. For those found by route 3, the difference in IRR between transgender persons AFAB and transgender persons AMAB was statistically significant (IRR = 12.66 *v.* 3.52, *p* < 0.001). After restricting the group of controls to those with a DTC at an iMHC, the IRRs associated with routes 2 and 3 dropped substantially, but were still of substantial magnitude for transgender persons AFAB. For transgender persons AMAB, the IRR was significantly lower than 1.00 (IRR = 0.60, 95%-CI 0.41 −0.86), but this may be caused by ‘overmatching’ and resulting underestimation (see above).

**Table 4.** Transgender persons in the Netherlands v. controls from the general population and (for route 2 and 3) controls from mental health care: numbers of persons, numbers of person-years of follow-up, numbers of Diagnosis Treatment Combinations for non-affective psychotic disorder (DTCs for NAPD, 2011–2019), Rates (number/ 10 000 person-years), and incidence rate ratios (IRRs), by route of identification (see Table 1), stratified by sex at birth: transgender persons assigned the male sex at birth (AMAB) and transgender persons assigned the female sex at birth (AFAB)

	Controls				Transgender persons						
	N	Number of person-years	Nr of DTCs for NAPD	Rate	N	Number of person-years	Nr of DTCs for NAPD	Rate	IRR	95%-confidence interval	AMAB v. AFAB by route:
	Controls from the general population										χ2, df, *p* value
Route					AMAB						
1	10 605	83 691	122	14.6	2121	17 308	43	24.8	1.70	1.20–2.41	
2	2290	18 921	17	9.0	458	3622	61	168.4	18.74	10.95–32.09	
3	3240	26 785	45	16.8	648	5409	32	59.2	3.52	2.24–5.54	
						route 1,2, v. 3: *χ*2, df, *p* value			53.90, 2	<0.001	
Route					AFAB						
1	8690	73 039	53	7.3	1738	14 914	29	19.5	2.68	1.70–4.21	2.42, 1, 0.12
2	1180	10 050	5	5.0	236	1846	31	167.9	33.74	13.12–86.78	1.12, 1, 0.29
3	1815	15 428	10	6.5	363	3046	25	82.1	12.66	6.08–26.36	8.47, 1, <0.001
						route 1,2, v. 3: *χ*2, df, *p* value			28.82, 2	<0.001	
Controls from mental health care											
Route					AMAB						
2	2290	18 290	202	110.4	458	3622	61	168.4	1.52	1.15–2.03	
3	3240	26 395	262	99.3	648	5409	32	59.2	0.60	0.41–0.86	
						route 1,2, v. 3: *χ*2, df, *p* value			20.52, 2	<0.001	
Route					AFAB						
2	1180	10 059	43	42.8	236	1846	31	167.9	3.93	2.47–6.23	11.65,1, <0.001
3	1815	15 507	60	38.7	363	3046	25	82.1	2.12	1.33–3.38	17.65,1, <0.001
						route 1,2, v. 3: *χ*2, df, *p* value			3.46, 2	0.1775	

More imporantly, the IRRs for transgender persons AFAB were significantly higher than the corresponding IRRs for transgender persons AMAB (Route 2: 3.93 *v.* 1.52, Route 3: 2.12 *v.* 0.60).

Similar results were found when data on dispensed antipsychotic medication were used to re-define the incident moment of treatment for NAPD (online Supplementary Table 2).

### Sequence of events

Online Supplementary Table 3 shows the frequency distribution for the time order of first registered indication(s) of transgender status and first registered indication of NAPD treatment (that is, a DTC for NAPD or, if applicable, the dispensing of antipsychotic medication within the period of 5 years before the first DTC for NAPD) for transgender persons, by route of selection.

A substantial number of subjects selected by routes 1 and 3 were diagnosed with NAPD after the first registration of data indicative of transgender status (50.0% and 45.6%, respectively). For those selected by route 2, the diagnosis of NAPD was often established before or just about the same time of the diagnosis of GID (83.7%).

## Discussion

This national population-based register study found a significantly higher risk of NAPD for transgender persons than for age- and sex-matched controls from the general population.

The data identified a large group of transgender persons who had received a diagnosis of a GID/GD at an iMHC but were not on a pathway towards gender-affirmative medical care (route 2). For this group the highest risks of NAPD were found.

### Strengths and limitations

We used national registries with well-defined data on demographic characteristics and health care utilisation that made it possible to determine the likely presence of transgender status. Since we used several selection routes, the study was not restricted to one specific clinic or setting. However, there are a number of limitations.

First, the data sources did not cover identical time periods. Thus, transgender persons identified by route 1 may have received a diagnosis of GID/GD from an iMHC before 2011 or in the years 2017–2019, and, thus, may actually belong to route 3. Those identified by route 2, in contrast, probably did not receive any gender-affirmative care during the years included in the study, as the data on administrative change of legal sex and hormonal therapy covered much longer periods of time than that for treatment at an iMHC. Given the strong association between NAPD and a diagnosis of GID/GD from an iMHC, the IRR for NAPD for route 1 may have been a little overestimated, as a number of persons actually belonging to route 3 were misclassified as belonging to route 1. As a consequence, the difference in IRR between route 1 on the one hand and routes 2 and 3 on the other may have been underestimated.

Second, the first registered diagnosis of NAPD during the period 2011–2019 does not necessarily indicate the first life-time episode, because some individuals may have received this diagnosis in earlier years. Thus, the estimated incidence rates and IRRs of NAPD reflect a mix of incident and prevalent cases, which may hamper a clear interpretation from an aetiological perspective. This mix of incident and prevalent cases together with a clear overrepresentation of younger age groups explains the relatively high incidence figures among controls when compared to, for example, the population-based figures reported by the recent European Network of National Schizophrenia Networks Studying Gene-Environment Interaction (EU-GEI) study (Jongsma *et al*. 2018). However, as the analysis was adjusted for actual follow-up time between 2011 and 2019, over- or underestimation of NAPD risk in favour of one group or the other is unlikely. Moreover, analyses that took dispensing of antipsychotic medication prior to the registered DTC for NAPD into account or restricted the study group to those under de age of 25 years yielded similar results. Still, the IRRs of this study should be interpreted with some caution as they do not reflect a ratio of lifetime incidence figures. Moreover, our outcome definition was based on diagnoses made by the responsible psychiatrist and these diagnoses were often not based on a semi-structured diagnostic interview.

Third, we cannot rule out the presence of patients with gender-related delusions who are misdiagnosed with GID/GD (À Campo & Nijman, 2016; À Campo *et al*. 2003). However, these delusions are closely associated with psychotic episodes and can often easily be differentiated from a genuine transgender status, that is mostly persistent from young childhood onwards. We expect that psychiatrists will be very careful and conservative when diagnosing GID/GD, the more for those known with a psychotic disorder (Schwarz *et al*. 2016).

Fourth, we cannot rule out the possibility that a small number of persons in our study group, perhaps in route 1, were diagnosed with a somatic disorder of sex development (DSD). However, most persons with DSD are patients with Klinefelter syndrome, Turner syndrome or congenital adrenal hyperplasia (de Vries *et al*. 2019). These disorders are generally not associated with the use of hormones opposite to the sex assigned at birth or an administrative change in legal sex. Thus, we may assume that the specificity of information on the use of cross-sex hormones, legal sex change, and a diagnosis of GID/GD for identifying transgender persons is high (Rich *et al*. 2021). On the other hand, a number of transgender persons who do not attend mental health care services or do not take steps to gender-affirmative medical care will have been missed by our algorithm. If these transgender persons have a less severe level of gender dysphoria and a lower psychosis risk, this may imply that the IRRs in our study were a little overestimated. On the other hand, this misclassification can also lead to an underestimation of the IRRs as the control group may include wrongly a number of transgender persons and, thus, the contrast between the groups becomes smaller. Of note, our data are not suitable to identify those with a non-binary gender identity. If assigned a DSM diagnosis, this may be ‘GID not otherwise specified’ (code 302.6). Fifth, the IRR of psychosis for those identified by route 2 may have been seriously overestimated, as such an extremely high IRR may be found for any group recruited at an institute for iMHC and compared to the general population. Although the selection was based on a diagnosis of GID/GD, it is likely that seeking treatment for psychotic symptoms was for some individuals the main reason for contacting an iMHC. As a consequence, this may lead to an overestimation of the IRR when we compare them to controls from the general population, including those who do not need any psychiatric treatment at all. For this reason, we selected a second group of controls with a record at an iMHC. The IRR for route 2 was still significantly higher than 1.00, which shows that, even within the segment of the population with psychiatric treatment, transgender status is significantly associated with the presence of psychosis.

Sixth, we could not relate the development of NAPD to the phases of gender-affirmative care. Data on gender-affirmative surgery, for instance, were not available. Future research with more fine-grained data is needed to examine the risk of psychosis in relation to the phases of gender-affirmative care (Simonsen *et al*. 2016).

Seventh, age, sex at birth, and (parental) country of birth were taken into account in the matching strategy. Other important co-variables, such as socio-economic status and substance abuse, were not included in the analysis. Future studies are needed to examine their impact on the causal pathway from transgender status to NAPD.

### Interpretation

The high risk of psychosis among transgender persons in general is in accordance with our postulated influence of minority stress and social defeat (Selten *et al*. 2013). As there are large differences in social status within the groups mentioned in the introduction, one could demand exposure measurement at the individual level before drawing conclusions about a role of social defeat. Transgender persons, however, are very often in a disadvantaged social and socio-economic position, and experience high levels of discrimination, stigmatisation, and social exclusion (Kuyper & Vanden Berghe, 2017). This highlights the important notion that prejudices, stereotypes and aggression towards people who do not comply with certain societal norms (such as cis-normativity) may have serious consequences for their mental health (Tan, Treharne, Ellis, Schmidt, & Veale, 2020). The long-term experience of social defeat (that is, outsider status or subordinate position) could contribute to the aetiology of NAPD by increasing dopamine activity in the striatum. In a previous study we showed that non-psychotic young adults with hearing impairment (who experience high levels of social exclusion) showed a greater amphetamine-induced dopamine release in the striatum than normal-hearing controls (Gevonden *et al*. 2014). However, the evidence of studies on dopaminergic functioning in non-psychotic individuals exposed to defeat is mixed: positive findings for migrants and for individuals exposed to childhood trauma and negative findings for individuals with autism and for those exposed to several stressors [for a list of references, see (Schalbroeck *et al*. 2021)].

The presence of a substantial number of individuals with a diagnosis of GID/GD and a treatment record at an iMHC, but without evidence of gender-affirmative treatment suggests that restrictions in providing this treatment for reasons of co-occuring psychiatric morbidity involve many transgender persons. However, this group may include individuals who also will benefit from this treatment (Meijer *et al*. 2017). It is also possible that a number of iMHC-identified transgender persons do not wish a (full) medical transition or are still exploring their gender issues. Future studies should further explore the treatment trajectories of transgender persons within mental health care: which diagnoses are made before and after the diagnosis of GID/GD, and what are the attitudes of mental health professionals towards gender-affirmative medical care for patients with a severe mental illness? Restricting care for other reasons, such as insurance denials, lack of competent providers or socio-economic factors are probably less relevant for transgender persons in the Netherlands, as all citizens are obliged by law to have medical insurance, and there is no distinction between public and private health-insurance companies.

A striking finding was the consistently higher IRRs for transgender persons AFAB compared to transgender persons AMAB. Since oestrogens appear to reduce the risk of NAPD, one could suggest that the use of oestrogens explains the lower IRR for transgender persons AMAB (Brand, de Boer, & Sommer, 2021; Riecher-Rossler, Butler, & Kulkarni, 2018). However, the IRR was also lower for transgender persons AMAB identified using route 2, thus, for those who did not use oestrogens. Further research is needed to substantiate our results and to find out which social and biological factors underlie the differences in IRRs.

## Conclusion

This study confirms and extends previous findings of increased risks of psychosis for transgender persons and supports the social defeat hypothesis of psychosis. The results also show the presence of a substantial number of transgender persons with severe psychiatric problems who have not (yet) taken steps to gender-affirmative care.

## Supporting information

Termorshuizen et al. supplementary material Termorshuizen et al. supplementary material

## Supplementary material

The supplementary material for this article can be found at https://doi.org/10.1017/S0033291723002088.

## References

[ref1] À Campo, J. M., & Nijman, H. (2016). Gender dysphoria and psychiatric symptoms. The Journal of Nervous and Mental Disease, 204(7), 558. doi:10.1097/NMD.0000000000000546. 27362702

[ref2] À Campo, J. M., Nijman, H., Merckelbach, H., & Evers, C. (2003). Psychiatric comorbidity of gender identity disorders: A survey among Dutch psychiatrists. American Journal of Psychiatry, 160(7), 1332–1336. doi:10.1176/appi.ajp.160.7.1332. 12832250

[ref3] Barr, S. M., Roberts, D., & Thakkar, K. N. (2021). Psychosis in transgender and gender non-conforming individuals: A review of the literature and a call for more research. Psychiatry Research, 306, 114272. doi:10.1016/j.psychres.2021.114272. 34808496

[ref4] Bazargan, M., & Galvan, F. (2012). Perceived discrimination and depression among low-income Latina male-to-female transgender women. BioMed Central Public Health, 12, 663. doi:10.1186/1471-2458-12-663. 22894701 PMC3497862

[ref5] Bolton, S. L., & Sareen, J. (2011). Sexual orientation and its relation to mental disorders and suicide attempts: Findings from a nationally representative sample. The Canadian Journal of Psychiatry, 56(1), 35–43. doi:10.1177/070674371105600107. 21324241

[ref6] Brand, B. A., de Boer, J. N., & Sommer, I. E. C. (2021). Estrogens in schizophrenia: Progress, current challenges and opportunities. Current Opinion in Psychiatry, 34(3), 228–237. doi:10.1097/YCO.0000000000000699. 33560022 PMC8048738

[ref7] Bresnahan, M., Begg, M. D., Brown, A., Schaefer, C., Sohler, N., Insel, B., … Susser, E. (2007). Race and risk of schizophrenia in a US birth cohort: Another example of health disparity? International Journal of Epidemiology, 36(4), 751–758. doi:10.1093/ije/dym041. 17440031

[ref8] Brown, G. R., & Jones, K. T. (2016). Mental health and medical health disparities in 5135 transgender veterans receiving healthcare in the veterans health administration: A case–control study. LGBT Health, 3(2), 122–131. doi:10.1089/lgbt.2015.0058. 26674598

[ref9] Castillejos, M. C., Martin-Perez, C., & Moreno-Kustner, B. (2018). A systematic review and meta-analysis of the incidence of psychotic disorders: The distribution of rates and the influence of gender, urbanicity, immigration and socio-economic level. Psychological Medicine, 48(13), 2101–2115. doi:10.1017/S0033291718000235. 29467052

[ref10] Cole, C. M., O'Boyle, M., Emory, L. E., & Meyer, W. J. (1997). Comorbidity of gender dysphoria and other major psychiatric diagnoses. Archives of Sexual Behavior, 26(1), 13–26. doi:10.1023/a:1024517302481. 9015577

[ref11] Coleman, E., Bockting, W., Botzer, M., Cohen-Kettenis, P., DeCuypere, G., Feldman, J., … Zucker, K. (2012). Standards of care for the health of transsexual, transgender, and gender- nonconforming people, version 7. International Journal of Transgenderism, 13(4), 165–232. doi:10.1080/15532739.2011.700873.

[ref12] de Freitas, L. D., Leda-Rego, G., Bezerra-Filho, S., & Miranda-Scippa, A. (2020). Psychiatric disorders in individuals diagnosed with gender dysphoria: A systematic review. Psychiatry and Clinical Neurosciences, 74(2), 99–104. doi:10.1111/pcn.12947. 31642568

[ref13] de Vries, A. L. C., Roehle, R., Marshall, L., Frisen, L., van de Grift, T. C., & Kreukels, B. P. C., … dsd-LIFE Group. (2019). Mental health of a large group of adults with disorders of sex development in six European countries. Psychosomatic Medicine, 81(7), 629–640. doi:10.1097/PSY.0000000000000718. 31232913 PMC6727927

[ref14] Dhejne, C., Lichtenstein, P., Boman, M., Johansson, A. L., Langstrom, N., & Landen, M. (2011). Long-term follow-up of transsexual persons undergoing sex reassignment surgery: Cohort study in Sweden. PLoS One, 6(2), e16885. doi:10.1371/journal.pone.0016885. 21364939 PMC3043071

[ref15] Dhejne, C., Van Vlerken, R., Heylens, G., & Arcelus, J. (2016). Mental health and gender dysphoria: A review of the literature. International Review of Psychiatry, 28(1), 44–57. doi:10.3109/09540261.2015.1115753. 26835611

[ref16] Dragon, C. N., Guerino, P., Ewald, E., & Laffan, A. M. (2017). Transgender medicare beneficiaries and chronic conditions: Exploring fee-for-service claims data. LGBT Health, 4(6), 404–411. doi:10.1089/lgbt.2016.0208. 29125908 PMC5731542

[ref17] Gevonden, M., Booij, J., van den Brink, W., Heijtel, D., van Os, J., & Selten, J. P. (2014). Increased release of dopamine in the striata of young adults with hearing impairment and its relevance for the social defeat hypothesis of schizophrenia. JAMA Psychiatry, 71(12), 1364–1372. doi:10.1001/jamapsychiatry.2014.1325. 25271822

[ref18] Gomez-Gil, E., Trilla, A., Salamero, M., Godas, T., & Valdes, M. (2009). Sociodemographic, clinical, and psychiatric characteristics of transsexuals from Spain. Archives of Sexual Behavior, 38(3), 378–392. doi:10.1007/s10508-007-9307-8. 18288600

[ref19] Hanna, B., Desai, R., Parekh, T., Guirguis, E., Kumar, G., & Sachdeva, R. (2019). Psychiatric disorders in the U.S. transgender population. Annals of Epidemiology, 39, 1–7. doi:10.1016/j.annepidem.2019.09.009. 31679894

[ref20] Haraldsen, I. R., & Dahl, A. A. (2000). Symptom profiles of gender dysphoric patients of transsexual type compared to patients with personality disorders and healthy adults. Acta Psychiatrica Scandinavica, 102(4), 276–281. doi:10.1034/j.1600-0447.2000.102004276.x. 11089727

[ref21] Hepp, U., Kraemer, B., Schnyder, U., Miller, N., & Delsignore, A. (2005). Psychiatric comorbidity in gender identity disorder. Journal of Psychosomatic Research, 58(3), 259–261. doi:10.1016/j.jpsychores.2004.08.010. 15865950

[ref22] Heylens, G., Elaut, E., Kreukels, B. P., Paap, M. C., Cerwenka, S., Richter-Appelt, H., … De Cuypere, G. (2014). Psychiatric characteristics in transsexual individuals: Multicentre study in four European countries. The British Journal of Psychiatry, 204(2), 151–156. doi:10.1192/bjp.bp.112.121954. 23869030

[ref23] Jongsma, H. E., Gayer-Anderson, C., Lasalvia, A., Quattrone, D., Mule, A., & Szoke, A. … European Network of National Schizophrenia Networks Studying Gene-Environment Interactions Work Package 2 (EU-GEI WP2) Group (2018). Treated incidence of psychotic disorders in the multinational EU-GEI study. JAMA Psychiatry, 75(1), 36–46. doi:10.1001/jamapsychiatry.2017.3554. 29214289 PMC5833538

[ref24] Khandaker, G. M., Barnett, J. H., White, I. R., & Jones, P. B. (2011). A quantitative meta-analysis of population-based studies of premorbid intelligence and schizophrenia. Schizophrenia Research, 132(2-3), 220–227. doi:10.1016/j.schres.2011.06.017. 21764562 PMC3485562

[ref25] Kuyper, L., & Vanden Berghe, W. (2017, May 1). Transgender personen in Nederland. Retrieved from https://repository.scp.nl/handle/publications/445.

[ref26] Linszen, M. M., Brouwer, R. M., Heringa, S. M., & Sommer, I. E. (2016). Increased risk of psychosis in patients with hearing impairment: Review and meta-analyses. Neuroscience & Biobehavioral Reviews, 62, 1–20. doi:10.1016/j.neubiorev.2015.12.012. 26743858

[ref27] Mazaheri Meybodi, A., Hajebi, A., & Ghanbari Jolfaei, A. (2014). Psychiatric axis I comorbidities among patients with gender dysphoria. Psychiatry Journal, 2014, 1–5. doi:10.1155/2014/971814. PMC4142737 25180172

[ref28] Meijer, J. H., Eeckhout, G. M., van Vlerken, R. H., & de Vries, A. L. (2017). Gender dysphoria and co-existing psychosis: Review and four case examples of successful gender affirmative treatment. LGBT Health, 4(2), 106–114. doi:10.1089/lgbt.2016.0133. 28170299

[ref29] Mirza, T., Taft, W., He, V. Y., Gooding, J., Dingwall, K., & Nagel, T. (2022). Incidence of treated first-episode psychosis amongst Aboriginal and non-Aboriginal youth in the Top End of the Northern Territory, Australia. Australasian Psychiatry, 30(4), 513–517. doi:10.1177/10398562221075193. 35196902

[ref30] Pellicane, M. J., & Ciesla, J. A. (2022). Associations between minority stress, depression, and suicidal ideation and attempts in transgender and gender diverse (TGD) individuals: Systematic review and meta-analysis. Clinical Psychology Review, 91, 102113. doi:10.1016/j.cpr.2021.102113. 34973649

[ref31] Petrovic-van der Deen, F. S., Cunningham, R., Manuel, J., Gibb, S., Porter, R. J., Pitama, S., … Lacey, C. (2020). Exploring indigenous ethnic inequities in first episode psychosis in New Zealand: A national cohort study. Schizophrenia Research, 223, 311–318. doi:10.1016/j.schres.2020.09.004. 32948382

[ref32] Post, D., & Veling, W., & Group investigators. (2021). Sexual minority status, social adversity and risk for psychotic disorders-results from the GROUP study. Psychological Medicine, 51(5), 770–776. doi:10.1017/S0033291719003726. 31875791 PMC8108393

[ref33] Rich, A. J., Poteat, T., Koehoorn, M., Li, J., Ye, M., Sereda, P., … Hogg, R. (2021). Development of a computable phenotype to identify a transgender sample for health research purposes: A feasibility study in a large linked provincial healthcare administrative cohort in British Columbia, Canada. Britisch Medical Journal Open, 11(3), e040928. doi:10.1136/bmjopen-2020-040928. PMC7996659 33766836

[ref34] Riecher-Rossler, A., Butler, S., & Kulkarni, J. (2018). Sex and gender differences in schizophrenic psychoses-a critical review. Archives of Women's Mental Health, 21(6), 627–648. doi:10.1007/s00737-018-0847-9. 29766281

[ref35] Schalbroeck, R., Termorshuizen, F., Visser, E., van Amelsvoort, T., & Selten, J. P. (2019). Risk of non-affective psychotic disorder or bipolar disorder in autism spectrum disorder: A longitudinal register-based study in the Netherlands. Psychological Medicine, 49(15), 2543–2550. doi:10.1017/S0033291718003483. 30460888

[ref36] Schalbroeck, R., van Velden, F. H. P., de Geus-Oei, L. F., Yaqub, M., van Amelsvoort, T., Booij, J., & Selten, J. P. (2021). Striatal dopamine synthesis capacity in autism spectrum disorder and its relation with social defeat: An [(18)F]-FDOPA PET/CT study. Translational Psychiatry, 11(1), 47. doi:10.1038/s41398-020-01174-w. 33441546 PMC7806928

[ref37] Schwarz, K., Fontanari, A. M., Mueller, A., Soll, B., da Silva, D. C., Salvador, J., … Lobato, M. I. (2016). Neural correlates of psychosis and gender dysphoria in an adult male. Archives of Sexual Behavior, 45(3), 761–765. doi:10.1007/s10508-015-0660-8. 26597648

[ref38] Selten, J. P., & Cantor-Graae, E. (2005). Social defeat: Risk factor for schizophrenia? The British Journal of Psychiatry, 187, 101–102. doi:10.1192/bjp.187.2.101. 16055818

[ref39] Selten, J. P., & Ormel, J. (2023). Low status, humiliation, dopamine and risk of schizophrenia. Psychological Medicine, 53(3), 609–613. doi:10.1017/S0033291722003816. 36695070 PMC9976000

[ref40] Selten, J. P., van der Ven, E., Rutten, B. P., & Cantor-Graae, E. (2013). The social defeat hypothesis of schizophrenia: An update. Schizophrenia Bulletin, 39(6), 1180–1186. doi:10.1093/schbul/sbt134. 24062592 PMC3796093

[ref41] Selten, J. P., van der Ven, E., & Termorshuizen, F. (2020). Migration and psychosis: A meta-analysis of incidence studies. Psychological Medicine, 50(2), 303–313. doi:10.1017/S0033291719000035. 30722795 PMC7083571

[ref42] Simonsen, R. K., Giraldi, A., Kristensen, E., & Hald, G. M. (2016). Long-term follow-up of individuals undergoing sex reassignment surgery: Psychiatric morbidity and mortality. Nordic Journal of Psychiatry, 70(4), 241–247. doi:10.3109/08039488.2015.1081405. 26479779

[ref43] Tan, K. K. H., Treharne, G. J., Ellis, S. J., Schmidt, J. M., & Veale, J. F. (2020). Gender minority stress: A critical review. Journal of Homosexuality, 67(10), 1471–1489. doi:10.1080/00918369.2019.1591789. 30912709

[ref44] Wiepjes, C. M., den Heijer, M., Bremmer, M. A., Nota, N. M., de Blok, C. J. M., Coumou, B. J. G., & Steensma, T. D. (2020). Trends in suicide death risk in transgender people: Results from the Amsterdam Cohort of Gender Dysphoria study (1972–2017). Acta Psychiatrica Scandinavica, 141(6), 486–491. doi:10.1111/acps.13164. 32072611 PMC7317390

