# Supplementary material for: The risk of psychosis for transgender individuals: a Dutch national cohort study
Source: Psychol Med. 2023 Aug 4;53(16):7923–32. doi: 10.1017/S0033291723002088 (PMC10755224; doi:10.1017/S0033291723002088)
Supplement: Termorshuizen et al. supplementary material [file S0033291723002088sup001.docx]

**Supplement eMethods**

Study group

Five controls from the general population were individually matched to a transgender person within strata defined by calendar year at birth in 5-years categories, sex at birth, country of origin, and migration status (1^st^ vs. 2^nd^ generation). The strata are shown below.

| Categories for birth year | Categories for country of origin | Categories for migration status |
| --- | --- | --- |
| >1950 - <1955 | - The Netherlands | - Native Dutch (both parents born in the |
| >1955 - <1960 | - Morocco | Netherlands) |
| >1960 - <1965 | - Turkey | - 1^st^ generation (born abroad) |
| >1965 - <1970 | - Surinam | - 2^nd^ generation (at least one parent born |
| >1970 - <1975 | - The Netherlands Antilles | abroad) |
| >1975 - <1980 | - non-Western: sub-Saharan Africa |  |
| >1980 - <1985 | - non-Western: Other ^1^ |  |
| >1985 - <1990 | - Western: Eastern Europe ^2^ |  |
| >1995 - <2000 | - Western: Other countries |  |
| >2000 - <2005 |  |  |
| >2005 - <2010 |  |  |
| >2010 |  |  |

^1^ including countries of the former Soviet Union with a predominantly Islamic religion, Japan, and Indonesia

^2^ including countries of the former Soviet Union with a predominantly Christian religion

Supplement Table 1. Transgender persons in the Netherlands vs. controls from the general population and (for route 2 and 3) controls from mental health care: numbers of persons, numbers of person-years of follow-up, numbers of Diagnosis Treatment Combinations for Non-Affective Psychotic Disorder (DTCs for NAPD, 2011-2019), after adjustment for dispensing of antipsychotic medication in the 5 years before the first registered DTC for NAPD, Rates (number/ 10,000 person-years), and Incidence Rate Ratios (IRRs), by route of selection (see Table 1 and Table 3)

|  | Controls | | |  |  | Transgender persons | | |  |  |  |
| --- | --- | --- | --- | --- | --- | --- | --- | --- | --- | --- | --- |
|  | N | Number of person-years | Nr of DTCs for NAPD | Rate |  | N | Number of person-years | Nr of DTCs for NAPD | Rate |  | IRR [95%-CI] |
|  | Controls from general population | | |  |  |  |  |  |  |  |  |
| Route |  |  |  |  |  |  |  |  |  |  |  |
| 1 | 19,219 | 156,569 | 99 | 6.32 |  | 3,831 | 32,128 | 44 | 13.70 |  | 2.17 [1.52-3.09] |
| 2 | 3,463 | 28,955 | 15 | 5.18 |  | 649 | 5,357 | 47 | 87.74 |  | 16.94 [9.47 – 30.29] |
| 3 | 5,026 | 42,151 | 26 | 6.17 |  | 983 | 8,371 | 29 | 34.64 |  | 5.62 [3.31-9.54] |
|  |  |  |  |  |  |  | route 1,2, vs 3: χ2, df, P value | | |  | 36.62, 2, <0.001 |
|  |  |  |  |  |  |  |  |  |  |  |  |
|  |  |  |  |  |  |  |  |  |  |  |  |
|  | Controls from mental health care | | |  |  |  |  |  |  |  |  |
| Route |  |  |  |  |  |  |  |  |  |  |  |
| 2 | 3,337 | 28,101 | 112 | 39.86 |  | 649 | 5,357 | 47 | 87.74 |  | 2.20 [1.57 – 3.09] |
| 3 | 4,911 | 41,594 | 178 | 42.79 |  | 983 | 8,371 | 29 | 34.64 |  | 0.81 [0.55 – 1.20] |
|  |  |  |  |  |  |  | route 1,2, vs 3: χ2, df, P value | | |  | 17.65, 2, <0.001 |
|  |  |  |  |  |  |  |  |  |  |  |  |

Supplement Table 2. Transgender persons in the Netherlands vs. controls from the general population and (for route 2 and 3) controls from mental health care: numbers of persons, numbers of person-years of follow-up, numbers of Diagnosis Treatment Combinations for Non-Affective Psychotic Disorder (DTCs for NAPD, 2011-2019), after adjustment for dispensing of antipsychotic medication in the 5 years before the first registered DTC for NAPD, Rates (number/ 10,000 person-years), and Incidence Rate Ratios (IRRs), by route of selection (see Table 1), stratified by sex at birth: transgender persons assigned the male sex at birth (AMAB) and transgender persons assigned the female sex at birth (AFAB)

|  | Controls | | |  |  | Transgender persons | | |  |  |  |  |  |
| --- | --- | --- | --- | --- | --- | --- | --- | --- | --- | --- | --- | --- | --- |
|  | N | Number of person-years | Nr of DTCs for NAPD | Rate |  | N | Number of person-years | Nr of DTCs for NAPD | Rate |  | IRR [95%-CI] | AMAB vs. AFAB by route: |  |
|  | Controls from general population | | |  |  |  |  |  |  |  |  | χ2, df, P value |  |
| Route |  |  |  |  |  | AMAB | |  |  |  |  |  |  |
| 1 | 10,553 | 83,587 | 70 | 8.37 |  | 2,105 | 17,257 | 27 | 15.65 |  | 1.87 [1.20-2.91] |  |  |
| 2 | 2,286 | 18,908 | 13 | 6.88 |  | 429 | 3,543 | 32 | 90.33 |  | 13.14 [6.90-25.03] |  |  |
| 3 | 3,218 | 26,735 | 23 | 8.60 |  | 635 | 5,374 | 19 | 35.35 |  | 4.11 [2.24-7.55] |  |  |
|  |  |  |  |  |  |  | route 1,2, vs 3: χ2, df, P value | | |  | 24.05, 2, <0.001 |  |  |
| Route |  |  |  |  |  | AFAB | |  |  |  |  |  |  |
| 1 | 8,666 | 72,982 | 29 | 3.97 |  | 1,726 | 14,871 | 17 | 11.43 |  | 2.88 [1.58-4.24] | 1.29, 1, 0.26 |  |
| 2 | 1,177 | 10,047 | 2 | 1.99 |  | 220 | 1,814 | 15 | 82.67 |  | 41.53 [9.50-181.6] | 1.96, 1, 0.16 |  |
| 3 | 1,808 | 15,416 | 3 | 1.95 |  | 348 | 2,997 | 10 | 33.37 |  | 17.15 [4.72-62.30] | 3.85, 1, 0.0496 |  |
|  |  |  |  |  |  |  | route 1,2, vs 3: χ2, df, P value | | |  | 14.66, 2, <0.001 |  |  |
|  | Controls from mental health care | | |  |  |  |  |  |  |  |  |  |  |
| Route |  |  |  |  |  | AMAB | |  |  |  |  |  |  |
| 2 | 2,181 | 18,095 | 93 | 51.40 |  | 429 | 3,543 | 32 | 90.33 |  | 1.76 [1.18-2.63] |  |  |
| 3 | 3,123 | 26,145 | 145 | 55.46 |  | 635 | 5,374 | 19 | 35.35 |  | 0.64 [0.40-1.03] |  |  |
|  |  |  |  |  |  |  | route 1,2, vs 3: χ2, df, P value | | |  | 13.17, 2, <0.001 |  |  |
| Route |  |  |  |  |  | AFAB | |  |  |  |  |  |  |
| 2 | 1,156 | 10,006 | 19 | 18.99 |  | 220 | 1,814 | 15 | 82.67 |  | 4.35 [2.21-8.57] | 5.10, 1, 0.02 |  |
| 3 | 1,788 | 15,449 | 33 | 21.36 |  | 348 | 2,997 | 10 | 33.37 |  | 1.56 [0.77-3.17] | 4.23, 1, 0.04 |  |
|  |  |  |  |  |  |  | route 1,2, vs 3: χ2, df, P value | | |  | 4.25, 2, 0.12 |  |  |
|  |  |  |  |  |  |  |  |  |  |  |  |  |  |

Supplement Table 3. Transgender persons with a Diagnosis Treatment Combination for Non-Affective Psychotic Disorder (DTC NAPD): interval between the first DTC NAPD (or first dispensing of antipsychotic medication, if any, within period of 5 years prior to this DTC) and the earliest registration of data indicative of transgender status, by route of selection

|  | Route 1 | Route 2 | Route 3 |  |
| --- | --- | --- | --- | --- |
| Time first DTC NAPD minus time first data on being transgender | N=72 | N=92 | N=57 |  |
| < -2 years | 26 (36.1%) | 43 (46.7%) | 15 (26.3%) |  |
| -2 - < 1 years | 10 (13.9%) | 34 (37.0%) | 16 (28.1%) |  |
| >1 years | 36 (50.0%) | 15 (16.3%) | 26 (45.6%) |  |
| Route 1,2, vs 3: χ2, df, P value |  |  | 29.955, 4, <0.001 |  |
|  |  |  |  |  |
| Mean interval in years (SD) | 0.1 (6.37) | -2.34 (3.17) | -0.7 (3.99) |  |
| Median interval in years | 0.5 | -2 | 0 |  |
|  |  |  |  |  |
